# Anaesthesia in a diagnosed ventricular septal defect with Guillain-Barré paediatric patient for videoassisted thoracic surgery

**DOI:** 10.4103/0019-5049.68389

**Published:** 2010

**Authors:** Gaurav S Tomar, Ashish Sethi, TC Kriplani, Shankar Agrawal

**Affiliations:** Department of Anaesthesiology and Critical Care, N.S.C.B. Medical College, Jabalpur (M.P.), India

**Keywords:** Guillain-Barre syndrome, paediatric anaesthesia, video-assisted thoracic surgery

## Abstract

Guillain-Barré syndrome with ventricular septal defect is rare finding. Delayed diagnosis, often leading to increased complications. This report describes an Guillain-Barré syndrome case and the special approaches required during anaesthesia. 4 yrs old male pt with Guillain-Barré syndrome diagnosed at time of ward admission, submitted to video-assisted thoracic surgery under uneventful general anaesthesia with sevoflurane, without neuromuscular blockers. The case highlights the frequency with which this syndrome so important for anaesthetic practice is diagnosed, adverse events, the best choice for the anaesthetic team and complications of pediatric Guillain-Barré syndrome.

## INTRODUCTION

Guillain-Barré syndrome with ventricular septal defect (VSD) is a rare finding. Delayed diagnosis often leads to increased complications. This report describes a Guillain-Barré syndrome case and the special approaches required during anaesthesia.

The case highlights the frequency with which this syndrome is so important for anaesthetic practice and complications of paediatric Guillain-Barré syndrome.

## CASE REPORT

A four-year-old male patient with Guillain-Barré syndrome with VSD diagnosed eventually after gastrointestinal infection was admitted due to recurrent URTI, tachypnoea, repetitive episodes of bronchopneumonia, bronchospasms and quadriparesis. On examination, bilateral coarse crepitation, pleural effusion and pansystolic murmur was heard at left sternal border.

**Figure 1:**
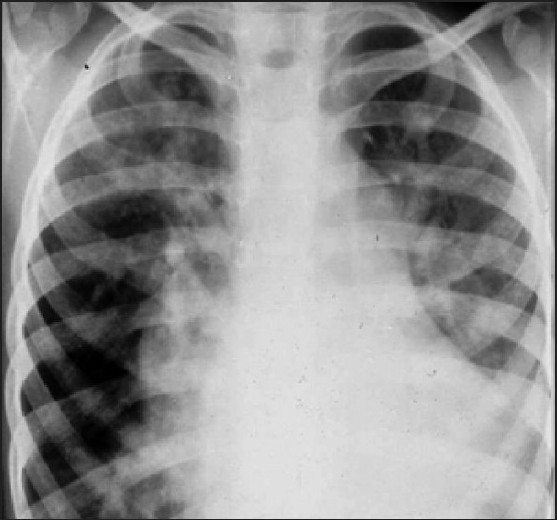
Chest X-ray of the patient

The idea was to perform VATS (video-assisted thoracic surgery) under general anaesthesia to decrease bronchopneumonia and pleural effusion episodes. Patient was admitted to the intensive care unit under mechanical ventilation and treated with IV-gamma globulin, netilmicin and meropenem for two weeks. Patient was transferred to the operating room under all aseptic conditions and on examination, patient was tachycardic with PR = 140 bpm, BP = 90/60 mmHg, RR = 32/min and chest X-ray [Figure 1] 2D and colour echocardiography revealing mild ventricular septal defect (<0.5 cm^2^).

Monitoring was done with capnography, pulse oximetry, invasive blood pressure and continuous ECG inside operating room.

Pre-medication was done with IM-glycopyrrolate (0.2 mg) 45 minutes before and intra-nasal midazolam puffs in each nostril 10 minutes before induction.

Patient was induced under Jackson Ree’s modification of Ayre’s t-piece circuit with sevoflurane and maintained on O_2_:N_2_O(40 : 60) and a combination of propofol infusion at 50μg/kg/min and remifentanil infusion at 0.25μg/kg/min.

Meanwhile, on lightning of anaesthesia, we required deepening with bolus propofol and remifentanil along with short acting b-blocker esmolol 0.1 mg/kg. Intravenous fluid (ringer lactate) administration was judged appropriately. Surgery was performed uneventfully and lasted 2 h without intercurrences. Patient was referred to the ICU for post-operative care.

Patient was regularly monitored for SpO_2_, temperature, pulse, invasive blood pressure and ECG. Tracheostomy was performed under local anaesthesia. After regular chest physiotherapy and proper treatment in ICU for two weeks, when found stable, the tubal drains were removed and when found capable of maintaining vitals including O_2_ saturation on air, he was discharged to the paediatric ward.

We decided for pre-medication with intranasal midazolam with 2–4 atomiser puffs in each nostril due to anxiety and with intramuscular glycopyrrolate because subsequent tachycardia associated with intravenous route may worsen haemodynamic stability. Since the VSD was small (<0.5 cm^2^), accompanied by a small pulmonary to systemic flow (<1.5/1.0) shunt, it did not cause significant haemodynamic derangement. A small VSD with high-resistance to flow permits only a small left-to-right shunt. The only danger in such associated VSD is the endocarditis-induced bacteraemia for which patient needs proper antibiotic prophylaxis.

As there was already diaphragm involvement (on mechanical ventilation), there has been no concern with respiratory depression.

## DISCUSSION

Combination infusion of propofol and remifentanil needs to be preferred modality to carry out such case as neuromuscular blockers may worsen symptoms.[1,2] Succinylcholine should not be used due to hyperkalaemia.[1,2] Guillain-Barré patients have increased number of extra-junctional acetylcholine receptors that allow further action and potassium release, which is not prevented with pre-curarisation.[2,3] There is also autonomic nervous system dysfunction and exacerbation.[3] There may be hypertensive crisis, tachycardia and other arrhythmias, making succinylcholine a bad choice.[1–3] Since this is a demyelinising polyradiculoneuritis, these patients are sensitive to non-depolarising muscle relaxant, which should be avoided for having their action time increased.[4,5]

Sevoflurane was the inhalational anaesthetic of choice for induction because of its faster induction and recovery, muscle relaxing action, low metabolism rate and adequate autonomic nervous system protection.

Guillain-Barré patients should be carefully followed-up and, when early diagnosis is confirmed effective treatment should be achieved with IV immunoglobulins.[6]

When patient is to be submitted to surgical procedure under anaesthesia, the case should be evaluated to decide the choice according to existing conditions and complications.
